# Primary care to further improve vertical HIV programming outcomes: From spillover to strategy

**DOI:** 10.1371/journal.pmed.1004434

**Published:** 2024-08-05

**Authors:** Elvin H. Geng, Ana Mocumbi, Wilbroad Mutale, Victor Davila-Roman, Michael Reid

**Affiliations:** 1 Washington University in St. Louis, St. Louis, Missouri, United States of America; 2 Universidade Eduadrdo Mondlane, Maputo, Mozambique; 3 University of Zambia, Lusaka, Zambia; 4 University of California San Francisco, San Francisco, California, United States of America; 5 PEPFAR, Bureau of Global Health Security and Diplomacy, US State Department National Public Health Institute, Washington DC, United States of America

*Pinto and colleagues find that growth of the community-based primary care led to reductions in both HIV incidence and AIDS-related mortality in Brazil. These findings suggest that primary care services can further advance and sustain gains achieved by successful vertical HIV services—with implications globally*.

As primary health care (PHC) systems grow and many countries are achieving or even exceeding 95-95-95 UNAIDS targets (e.g., Botswana), many HIV programs around the world—particularly those with donor-funded and verticalized services—face a strategic crossroads. Should investments to address HIV maintain a strict focus on HIV control targets, or should these investments be seen as strategies to also strengthen health systems and thereby grow primary care capacity? While recent clinical trial data has demonstrated that integrating HIV care with hypertension and diabetes management in Africa did not undermine HIV outcomes [1], there remains little real-world data about the effects of primary care systems on HIV outcomes where successful vertical HIV programs already exist [2]. Evidence that primary care can actually improve HIV outcomes could push objectives of HIV funding away from a sole focus on HIV epidemic targets toward supporting, where possible, health systems. In essence, this approach would allow “positive spillover” of HIV programming to become an intentional strategy, accompanied by measures of progress in health systems infrastructure and function. To date, much of the success of the global response to HIV in some of the hardest-hit regions has been due to focused advocacy, disease-specific funding (e.g., the US President’s Emergency Plan for AIDS Relief), and verticalized service delivery. HIV exceptionalism may well have been the secret to success to date, but will it be the right strategy for sustaining this hard-won progress into the future?

In a new study, Priscila Pinto and colleagues evaluated the impact of the Brazilian Family Health Strategy (FHS), a PHC program, on AIDS incidence and mortality among 3.4 million low-income Brazilians from 2007 to 2015 [3]. Brazil’s FHS is a community-oriented form of primary care composed of a team that includes a doctor, nurses, and health assistants who cover groups of about 3,500 residents [4]. At the time of the end of the study period (2016), the FHS employed approximately 265,000 community health workers (CHWs) in total and served nearly 67% of the entire population of the country. CHWs are integral to the FHS and are responsible for administrative activities such as registering families in their area and monitoring living conditions, as well as providing primary care through patient education and monitoring chronic conditions. Pinto and colleagues compared areas with 100% FHS coverage to areas with low or no FHS coverage and found that AIDS incidence and mortality rates were lower by 24% and 31%, respectively, in fully covered areas. The reductions were greater among individuals aged 35 years or older. FHS coverage has already been linked to numerous positive health outcomes, including more accurate mortality statistics, improved breastfeeding rates, decreased inequity in healthcare utilization, near 100% immunization uptake, reduced fertility rates, as well as improvements in infant mortality [5,6]. The study from Pinto and colleagues adds to this body of evidence in that it examines looks at effects of FHS for a condition that already enjoyed a substantial standalone system of care—HIV. The positive findings add to the impressive array of analyses already showing positive effects of the FHS.

To assess the implications of this work, we draw from concepts from evaluation research by Weiss [7], who noted that good evaluation can help public discourse in 3 ways: to *reconceptualize* a problem, to *mobilize* support for action, and to act as a *guidance*. Pinto and colleagues succeed on each of these 3 counts. Their study helps to reconceptualize the role of expanded primary care services for HIV programming. Instead of a competing investment, primary care services may instead represent a phase of the HIV response that is focused on sustainment and reach. This does not mean that epidemic targets should be dropped or that donor investments become directly focused on health systems, but in reality, investments in training health workers, infrastructure, and information systems for HIV are already leveraged to support other health services, and optimizing these effects can augment the value of vertical investments. Pinto and colleagues’ findings also mobilize new positions and alliances in the strategy of donor investments. If we no longer imagine that we have to choose between HIV services and health systems, the debate shifts, enabling new coalitions and alliances to form. Finally, this paper also offers guidance. Brazil’s primary care approach, which leans heavily on CHWs, is a remarkable example of the merging of public health and primary care. While the study did not include a comparison to other, less community-embedded models of primary care, the findings strongly suggest that a community engagement model is key. This is fortunate, as many HIV programs in high-prevalence countries already employ a range of community-based health workers, have experience with community engagement, and serve as a model for the wider primary care systems.

The top-line insights from the study by Pinto and colleagues are particularly relevant for high HIV-burden countries in Africa, many of which are looking to integrate HIV programs and primary health care as part of a sustainable health agenda. The demographic and economic differences between Brazil and other high HIV-burden countries notwithstanding, the study could inform the discourse on how HIV programming can yield dividends for people living with HIV as well as for the general population. In many settings, verticalized HIV treatment programs are nearing or at the apogee of what these programs can do when measured by HIV outcomes alone. Further progress may depend on a paradigm in which disease-specific investments are deployed to strengthen broader health system that functions in ways that complement vertical systems. As population morbidity and mortality from HIV declines, people living with HIV are increasingly susceptible to the same chronic diseases that confront those living without HIV, such as metabolic disorders, cardiovascular disease, and mental health conditions—conditions that are best managed with primary care. While not captured in the analysis by Pinto and colleagues, integrated services may also have other beneficial externalities specific for people living with and at risk for HIV, including but not limited to reducing stigma, optimizing the cost effectiveness of scarce resources, and ensuring delivery of holistic client-centered programming.

Despite the study’s clear findings, there are many unanswered questions that future studies must address. For example, Pinto and colleagues did not examine non-AIDS–related mortality in people living with HIV. Reductions in these outcomes (e.g., cardiovascular disease), especially important in older persons, would suggest that primary care is better able to address the rising contribution of noncommunicable diseases to mortality in people living with HIV. In addition, the observed association between FHS and reductions in HIV incidence implies that primary care improved HIV prevention. How did it do so? There are a number of plausible explanations. First, it could be that HIV prevention services were more successfully delivered through primary care mechanisms rather than via specific HIV programming. Primary care may be a more appropriate place for patients to receive prevention services when stigma limits the use of HIV services. Observed reductions in mortality invite similar speculation and also call for further research. Reductions in AIDS-related mortality that were observed in this study could have been mediated, for example, by reductions in late presentation if diagnosis and linkage to services were improved through primary care services. Primary care empanelment may also lead to improved outcomes for co-morbid diseases like hypertension and cardiovascular disease, as well as more consistent engagement in HIV services. Whether the findings from Brazil can be replicated in countries with growing primary care systems or where critical gains in HIV programs have not yet been secured is far from clear, but such further analyses of these data can help inform generalizability of findings from Brazil. In addition, a rising tide of ongoing implementation research, such as the Heart, Lung, and Blood Co-morbiditieS IMplementation Models in People Living with HIV (SIMPLE) network, will help to answer how management of noncommunicable diseases in individuals living with HIV can best be carried out.

In conclusion, the research from Pinto and colleagues contributes significantly to the evidence base advocating for integrated HIV programs within PHC systems. By demonstrating the tangible benefits of the FHS program in Brazil—considered to already have one of the vertical HIV programs in the world—the study underscores the importance of investment in PHC as a strategy for achieving an end to the HIV epidemic while enhancing the health and well-being of populations globally. The analysis suggests that the real question going into the future will not be about trade-offs between integrated PHC and dedicated services for people living with HIV. Instead, we should ask how investments in primary care can optimally advance the health of the whole person living with, and without, HIV.
